# Resting heart rate, cognitive function, and inflammation in older adults: a population-based study

**DOI:** 10.1007/s40520-023-02576-8

**Published:** 2023-10-29

**Authors:** Ming Mao, Rui Liu, Yi Dong, Chaoqun Wang, Yifei Ren, Na Tian, Shi Tang, Tingting Hou, Lin Cong, Yongxiang Wang, Yifeng Du, Chengxuan Qiu

**Affiliations:** 1 Department of Neurology, Cheeloo College of Medicine, Shandong Provincial Hospital, Shandong University, No. 324 Jingwuweiqi Road, Jinan, 250021 Shandong People’s Republic of China; 2 Department of Neurology, Shandong Provincial Hospital affiliated to Shandong First Medical University, Jinan, Shandong People’s Republic of China; 3 Medical Science and Technology Innovation Center, Shandong First Medical University & Shandong Academy of Medical Sciences, Jinan, 250021 Shandong People’s Republic of China; 4 Institute of Brain Science and Brain-Inspired Research, Shandong First Medical University & Shandong Academy of Medical Sciences, Jinan, Shandong People’s Republic of China; 5 Key Laboratory of Endocrine Glucose & Lipids Metabolism and Brain Aging in Shandong First Medical University, Ministry of Education of the People’s Republic of China, Jinan, Shandong People’s Republic of China; 6 Aging Research Center and Center for Alzheimer Research, Department of Neurobiology, Care Sciences and Society, Karolinska Institutet-Stockholm University, Stockholm, Sweden

**Keywords:** Resting heart rate, Cognitive function, Low-grade inflammation, Endothelial dysfunction, Population-based study

## Abstract

**Background:**

Emerging evidence has linked elevated resting heart rate (RHR) with poor cognitive function in older adults, but the mechanisms underlying their association are poorly understood.

**Methods:**

This population-based cross-sectional study included 4510 dementia-free participants (age ≥ 65 years; 56.9% females; 38.3% no formal education) in the baseline examination of the Multidomain Interventions to Delay Dementia and Disability in Rural China study. Of these, 1,386 had data on serum proinflammatory cytokines and adhesion molecules. RHR was measured using 12-lead electrocardiograph. We used the Mini-Mental State Examination (MMSE) and a neuropsychological test battery to assess cognitive function. Data were analyzed using the general linear and restricted cubic splines models.

**Results:**

People with high RHR were more likely to have cardiometabolic diseases and worse cognitive function (*p* < 0.05). There was an inverted J-shaped association of RHR with MMSE and attention scores. Having RHR ≥ 80 bpm (vs. 60–69 bpm) was significantly associated with the multivariable-adjusted β coefficients of − 0.58 [95% confidence interval (CI), − 1.00, − 0.16] for MMSE score and − 0.08 (− 0.15, − 0.01) for attention score. In the serum biomarker subsample, RHR was linearly associated with serum interleukin-6 (IL-6) (β coefficient = 0.19; 95%CI 0.14, 0.24), IL-8 (0.08; 0.02, 0.13), IL-10 (0.09; 0.04, 0.15), tumor necrosis factor-α (0.06; 0.01, 0.11), monocyte chemotactic protein-1 (0.09; 0.04, 0.15), intercellular adhesion molecule-1 (0.16; 0.11, 0.22), and vascular cell adhesion molecule-1 (0.11; 0.06, 0.16).

**Conclusions:**

There is an inverted J-shaped association of RHR with attention and global cognition. Poor cognitive function and high RHR may be linked through systemic low-grade inflammation and endothelial injury.

**Supplementary Information:**

The online version contains supplementary material available at 10.1007/s40520-023-02576-8.

## Introduction

Elevated resting heart rate (RHR) has been associated with major adverse cardiac events and cognitive dysfunction [1–5]. Cardiovascular disease (CVD) and cognitive impairment share common risk factors and pathophysiology, such as unhealthy lifestyles, metabolic risk factors, arterial stiffness, and cerebral microvascular disease [6–8]. Thus, it has been suggested that the heart, the brain, and cognitive function are tightly connected in the aging process [4, 9]. Evidence from pooled data of large-scale clinical trials has shown that elevated RHR is associated with accelerated cognitive decline among patients with high cardiovascular risk [10]. Besides, a community-based study found that high RHR in midlife was cross-sectionally related to poor global cognitive function among participants free of stroke and atrial fibrillation [5]. However, the association of RHR with specific cognitive domains (e.g., episodic memory, verbal fluency, attention, and executive function) in a general population of older adults has been rarely explored. Notably, a substantial proportion of people with low RHR (e.g., < 60 beats per minute, bpm) may have concomitant sinus node or conduction diseases [2, 4], which may lead to reduced craniocerebral perfusion and cognitive dysfunction. Therefore, it is essential to clarify the complex association of RHR with global and domain-specific cognitive function in a general population of older adults.

While a low RHR may be linked with cognitive dysfunction through cerebral hypoperfusion, the pathophysiological mechanism linking elevated RHR to cognitive function is poorly understood. It is proposed that elevated RHR may alter the mechanical stress on the blood vessel wall, and further result in oxidative stress, endothelial injury, atherosclerosis, and eventually plaque rupture [11]. In addition, systemic low-grade inflammation (LGI) and endothelial dysfunction (ED) play a vital role in the pathogenesis of CVD [12, 13]. LGI markers consist of proinflammatory cytokines (e.g., tumor necrosis factor alpha [TNF-α], IL-1β, IL-6, IL-8), and monocyte chemoattractant protein-1 [MCP-1]) and anti-inflammatory cytokines (e.g., IL-4 and IL-10), while ED markers include serum intercellular cell adhesion molecule-1 (ICAM-1) and vascular cell adhesion molecule-1 (VCAM-1) [14–16]. Systematic reviews have linked these biomarkers with blood–brain barrier breakdown, cerebral small vessel disease, and cognitive impairment [17–19]. Therefore, LGI and ED may represent important pathways linking elevated RHR with poor cognition. Previous studies showed that RHR was associated with a limited number of inflammatory biomarkers (e.g., IL-6 and high-sensitivity C reactive protein [hsCRP]) in middle-aged adults who were free of clinical CVD [20]. It remains to be elucidated that whether RHR is associated with biomarkers of LGI and ED in a general population of older adults.

Therefore, we hypothesized that abnormal RHR was associated with poor cognitive function, as well as increased serum biomarkers of LGI and ED in old age. In this population-based cross-sectional study, we aim to test this hypothesis by assessing the association of RHR with function of global cognition and multiple cognitive domains, and serum biomarkers of LGI and ED among rural-dwelling older adults in China.

## Methods

### Study design and participants

This is a population-based cross-sectional study. The study used data from the baseline assessments of the Multidomain INterventions to delay Dementia and disability in rural China (MIND-China) study, a participating project of the World-Wide FINGERS Network [21]. MIND-China targeted people who were aged ≥ 60 years and living in the 52 villages of Yanlou town, Yanggu County, western Shandong Province. In March-September 2018, a total of 5765 participants (74.9% of all eligible persons) underwent baseline examination. We excluded participants who were aged 60–64 years (n = 519) because they were substantially underrepresented of this age group due the fact that a considerable proportion of people in this age group were working as rural migrant workers, and thus, could not attend the assessments. Of the remaining 5246 participants who were aged ≥ 65 years, we further excluded 736 persons due to prevalent dementia (n = 302), severe mental health problems (e.g., depressive symptoms and schizophrenia, n = 46), or missing data on cognitive function (n = 144), RHR (n = 2), or covariates (n = 242), leaving 4510 participants for the analysis involving RHR in association with cognitive function (analytical sample 1). Compared to individuals in the analytical sample, those who were excluded due to missing data (n = 388) were older (mean age, 73.91 vs. 71.26 years, *p* < 0.001), less likely to be female (50.8% vs. 56.9%, *p* = 0.02), and less educated (no formal schooling, 46.6% vs. 38.3%, *p* = 0.005). Out of the 4510 participants, data on serum biomarkers of LGI and ED were available in a subsample of 1,386 participants, which were selected using the cluster (village)-randomized sampling approach. Supplementary Fig. S1 shows the flowchart of the study participants.

### Data collection and assessments

The trained medical staff collected data following the structured questionnaire via face-to-face interviews, clinical and neurological examination, neuropsychological assessments, and laboratory tests [21]. RHR was derived from the 12-lead CM300 electrocardiograph (Comen Corp., Shenzhen, Guangdong, China) in a resting supine position, and was categorized into < 60, 60–69 (reference), 70–79, and ≥ 80 beats per minute (bpm) [2, 4, 5], or used as a continuous variable. Education was categorized into no formal education, primary school (1–5 years), and middle school or above (≥ 6 years). Smoking status and alcohol intake were categorized into never, former, and current. Body mass index (BMI), systolic blood pressure, and diastolic blood pressure were measured as previous studies [21]. After an overnight fast, peripheral blood samples were collected. Total cholesterol and blood glucose were measured using an automatic biochemical analyzer (DIRUI CS-600B; DIRUI Corporation, Changchun, China). Estimated glomerular filtration rate (eGFR) was calculated following the creatinine equation from the Chronic Kidney Disease Epidemiology Collaboration [22]. Diabetes mellitus, dyslipidemia, and hypertension were defined by integrating self-reported history of respective disorders, clinical examination and blood tests (i.e., blood pressure measurement, fasting blood glucose, and serum lipids), and current use of respective mediations (i.e., antihypertensive, blood glucose-lowering, and lipids-lowering drugs), following the approaches as previously described [23]. Coronary heart disease, heart failure, and atrial fibrillation were defined as self-reported history of the disease or diagnosis by clinicians based on ECG or medical history. Stroke and transient ischemic attacks (TIAs) were ascertained by self-reported history or diagnosis by neurologists through clinical and neurological examination. We defined load of cardiovascular morbidity by counting the number of cardiovascular diseases that concurrently occurred in the same participant, including coronary heart disease, heart failure, atrial fibrillation, stroke, and TIAs. Antithrombotic agents (acetylsalicylic acid, clopidogrel, and warfarin) and cardiac agents (amiodarone, digoxin, propafenone, isosorbide mononitrate, glyceryl trinitrate, and trimetazidine) were considered as confounders because of their potential associations with both RHR and cognitive function [24, 25]. Information on the current use of medications was collected and classified according to the Anatomical Therapeutic Chemical (ATC) classification system, as previously reported [26]. Apolipoprotein E (*APOE*) genotype was detected using multiple-PCR amplification analysis and was dichotomized into carriers vs. non-carriers of the ε4 allele.

### Assessments of cognitive function

A neuropsychological test battery was administered to assess cognitive function, as previously described [21, 27]. In brief, we used both the Mini-Mental State Examination (MMSE) score and the global cognitive z-score to assess global cognitive function. We assessed the function of the following four cognitive domains: episodic memory (Auditory Verbal Learning Test-immediate recall, long-delayed free recall, and long-delayed recognition), verbal fluency (Verbal Fluency Test-categories of animals, fruits, and vegetables), attention (Digit Span Test forward and Trail Making Test A), and executive function (Digit Span Test-backward and Trail Making Test B). The raw test scores were transformed into z-scores. Given that all cognitive domains were assessed using multiple tests, we calculated the composite z-score for each of the cognitive domains by averaging the z-scores of the tests for that domain. A composite z-score for global cognitive function was computed as the mean of all z-scores for individuals with data in all four cognitive domains.

### Measurements of LGI and ED biomarkers

Peripheral blood samples were collected into procoagulant separating gel vacutainers, and then centrifuged, aliquoted, and stored at – 80 ℃ for future analysis. Serum cytokine assays were performed using the Meso Scale Discovery (Rockville, MD, USA) V-PLEX® Panel, which included IL-6, IL-8, IL-10, TNF-α, MCP-1, ICAM-1, and VCAM-1. For each plate, two quality control samples were carried out and the within-batch and inter-batch CVs were < 20%. Serum biomarkers of LGI included IL-6, IL8, IL-10, TNF-α, and MCP-1, and biomarkers of ED included ICAM-1 and VCAM-1, based on previous studies [14–16].

### Statistical analysis

We compared characteristics of the study participants by RHR strata using chi-squared test for categorical variables and Kruskal–Wallis test for continuous variables with non-normal distribution. We used the general linear regression models to assess the associations of RHR with cognitive score as well as serum LGI and ED biomarkers. In addition, we implemented the restricted cubic spline (RCS) models to investigate the non-linear associations of RHR with cognitive score.

We reported the main results from 2 models: Model 1 was adjusted for age, sex, and education; and Model 2 was additionally adjusted for smoking status, alcohol intake, BMI, dyslipidemia, hypertension, diabetes, estimated glomerular filtration rate, cardiovascular multimorbidity, *APOE* genotype, anti-thrombotic agents, and cardiac agents.

A two-tailed *p* < 0.05 was considered statistically significant. R version 3.6.2 (R Project for Statistical Computing; http://www.r-project.org) was used for all statistical analyses.

## Results

### Characteristics of study participants

The mean age of the 4,510 participants was 71.26 (age range, 65–93; standard deviation, 5.0) years, 56.9% were females, and 38.3% had no formal education. Compared to people with lower RHR, those with higher RHR were older, more likely to be female, and less educated; were less likely to smoke and drink alcohol; had higher levels of blood pressure and total cholesterol, and lower levels of eGFR, MMSE score, and cognitive z-scores; had high prevalence of diabetes, hypertension, dyslipidemia, coronary heart disease, atrial fibrillation, and heart failure; and were more likely to take cardiac agents (all *p* < 0.05, Table 1). In addition, BMI slightly differed across RHR groups, but did not show a linear trend with RHR.

**Table 1:** Characteristics of the study participants by resting heart rate levels (n = 4510)

Characteristics	Total sample	Resting heart rate, bpm				
	(n = 4510)	< 60 (n = 1041)	60–69 (n = 1837)	70–79 (n = 1072)	≥ 80 (n = 560)	*P*-value
Age, years	71.26 (5.0)	70.77 (4.7)	71.04 (4.9)	71.55 (5.1)	72.34 (5.4)	< 0.001
Female	2568 (56.9)	445 (42.7)	1059 (57.6)	725 (67.6)	339 (60.5)	< 0.001
Education						< 0.001
No formal education	1728 (38.3)	323 (31.0)	696 (37.9)	466 (43.5)	243 (43.4)	
Primary school	2028 (45.0)	516 (49.6)	835 (45.5)	443 (41.3)	234 (41.8)	
Middle school or above	754 (16.7)	202 (19.4)	306 (16.7)	163 (15.2)	83 (14.8)	
Smoking status						< 0.001
Never	2888 (64.0)	553 (53.1)	1179 (64.2)	781 (72.9)	375 (67.0)	
Former	683 (15.1)	179 (17.2)	271 (14.8)	132 (12.3)	101 (18.0)	
Current	939 (20.8)	309 (29.7)	387 (21.1)	159 (14.8)	84 (15.0)	
Alcohol intake						< 0.001
Never	2769 (61.4)	527 (50.6)	1138 (61.9)	746 (69.6)	358 (63.9)	
Former	429 (9.5)	122 (11.7)	145 (7.9)	84 (7.8)	78 (13.9)	
Current	1312 (29.1)	392 (37.7)	554 (30.2)	242 (22.6)	124 (22.1)	
BMI, kg/m^2^	24.89 (3.8)	24.88 (3.6)	25.00 (3.6)	24.90 (3.8)	24.52 (4.4)	0.04
SBP, mmHg*	144.02 (21.4)	143.72 (20.9)	142.99 (21.0)	144.65 (21.0)	146.70 (23.7)	0.004
DBP, mmHg*	85.03 (10.9)	83.59 (10.7)	84.37 (10.7)	86.20 (10.5)	87.66 (11.7)	< 0.001
TC, mmol/l	4.99 (1.0)	4.86 (0.9)	4.96 (1.0)	5.07 (1.0)	5.15 (1.1)	< 0.001
eGFR, (ml/min/1.73m^2^)	78.60 (13.0)	79.03 (12.2)	79.03 (13.0)	78.55 (13.0)	76.49 (14.1)	0.005
Diabetes	629 (13.9)	94 (9.0)	242 (13.2)	190 (17.7)	103 (18.4)	< 0.001
Hypertension	3034 (67.3)	707 (67.9)	1181 (64.3)	740 (69.0)	406 (72.5)	0.001
Dyslipidemia	1047 (23.2)	180 (17.3)	413 (22.5)	287 (26.8)	167 (29.8)	< 0.001
Number of CVD	0.42 (0.6)	0.43 (0.6)	0.39 (0.6)	0.41 (0.6)	0.55 (0.8)	0.002
Coronary heart disease	981 (21.8)	223 (21.4)	373 (20.3)	233 (21.7)	152 (27.1)	0.008
Atrial fibrillation	68 (1.5)	7 (0.7)	9 (0.5)	10 (0.9)	42 (7.5)	< 0.001
Heart failure	137 (3.0)	35 (3.4)	42 (2.3)	33 (3.1)	27 (4.8)	0.02
Stroke	674 (14.9)	170 (16.3)	272 (14.8)	155 (14.5)	77 (13.8)	0.49
TIA	51 (1.1)	10 (1.0)	22 (1.2)	11 (1.0)	8 (1.4)	0.83
Anti-thrombotic agents	302 (6.7)	63 (6.1)	125 (6.8)	74 (6.9)	40 (7.1)	0.81
Cardiac agents	74 (1.6)	14 (1.3)	25 (1.4)	17 (1.6)	18 (3.2)	0.02
MMSE score	20.99 (5.9)	21.72 (5.8)	21.23 (5.8)	20.43 (5.8)	19.92 (6.1)	< 0.001
Cognitive z-scores*						
Global cognition	− 0.23 (2.6)	0.02 (2.5)	− 0.12 (2.5)	− 0.47 (2.5)	− 0.64 (2.7)	< 0.001
Memory	− 0.05 (0.9)	− 0.02 (0.9)	− 0.01 (0.9)	− 0.09 (0.9)	− 0.15 (0.9)	0.002
Verbal fluency	− 0.03 (0.8)	0.01 (0.8)	0.01 (0.8)	− 0.09 (0.8)	− 0.10 (0.8)	< 0.001
Attention	− 0.07 (0.8)	0.01 (0.8)	− 0.05 (0.9)	− 0.11 (0.8)	− 0.22 (0.8)	< 0.001
Executive function	− 0.12 (0.9)	− 0.02 (0.9)	− 0.10 (0.9)	− 0.22 (0.9)	− 0.22 (0.9)	< 0.001
*APOE* ε4 allele	709 (15.7)	165 (15.9)	298 (16.2)	146 (13.6)	100 (17.9)	0.12

Data are mean (standard deviation) or n (%)

*RHR* resting heart rate, *BMI* body mass index, *SBP* systolic blood pressure, *DBP* diastolic blood pressure, *TC* total cholesterol, *eGFR* estimated glomerular filtration rate, *CVD* cardiovascular disease, *TIA* transient ischemic attack, *MMSE* Mini-Mental State Examination, *APOE*, apolipoprotein E gene

*The number of participants with missing values was 6 for SBP and DBP, 330 for global cognition score, 258 for memory score, 207 for verbal fluency score, 220 for attention score, and 257 for executive function score

### Associations of RHR with cognitive function

When RHR was analyzed as a categorical variable, participants with RHR ≥ 80 bpm (vs. 60–69 bpm) had the demographic-adjusted β coefficient of − 0.66 [95% confidence interval (CI) − 1.08, − 0.24] for MMSE score, − 0.24 (− 0.45, − 0.03) for global cognitive z-score, and − 0.10 (− 0.17, − 0.03) for attention z-score (Table 2, Model 1). Besides, RHR < 60 bpm was marginally associated with lower MMSE score and verbal fluency z-score. The results were virtually unchanged after further controlling for a wide range of potential confounders, except that the association of RHR ≥ 80 bpm with lower global cognitive z-score was attenuated and became statistically non-significant (Table 2, Model 2).

**Table 2:** Associations of resting heart rate with MMSE score and z-scores of global cognition, memory, verbal fluency, attention, and executive function

Resting heart rate	N^a^	β coefficient (95% confidence interval), cognitive score	
		Model 1^b^	Model 2^b^
MMSE score (n = 4510)			
RHR < 60 bpm	1837	− 0.32 (− 0.66, 0.02)	− 0.32 (-0.65, 0.02)
RHR 60–69 bpm	1041	0.00 (reference)	0.00 (reference)
RHR 70–79 bpm	1072	− 0.15 (− 0.48, 0.19)	− 0.10 (− 0.43, 0.23)
RHR ≥ 80 bpm	560	− 0.66 (− 1.08, − 0.24) ^†^	− 0.58 (− 1.00, − 0.16) ^†^
Global cognitive z-score (n = 4180)			
RHR < 60 bpm	1714	− 0.12 (− 0.29, 0.05)	− 0.10 (− 0.27, 0.07)
RHR 60–69 bpm	982	0.00 (reference)	0.00 (reference)
RHR 70–79 bpm	978	− 0.12 (− 0.29, 0.04)	− 0.10 (− 0.27, 0.07)
RHR ≥ 80 bpm	506	− 0.24 (− 0.45, − 0.03)*	− 0.17 (− 0.38, 0.05)
Memory z-score (n = 4252)			
RHR < 60 bpm	1739	− 0.01 (− 0.08, 0.05)	− 0.01 (− 0.07, 0.06)
RHR 60–69 bpm	993	0.00 (reference)	0.00 (reference)
RHR 70–79 bpm	1004	− 0.06 (− 0.13, 0.00)	− 0.06 (− 0.12, 0.01)
RHR ≥ 80 bpm	516	− 0.07 (− 0.15, 0.01)	− 0.05 (− 0.13, 0.03)
Verbal fluency z-score (n = 4303)			
RHR < 60 bpm	1762	− 0.05 (− 0.11, 0.01)	− 0.04 (− 0.10, 0.01)
RHR 60–69 bpm	1002	0.00 (reference)	0.00 (reference)
RHR 70–79 bpm	1016	− 0.05 (− 0.11, 0.00)	− 0.05 (− 0.10, 0.01)
RHR ≥ 80 bpm	523	− 0.04 (− 0.11, 0.03)	− 0.02 (− 0.09, 0.06)
Attention z-score (n = 4290)			
RHR < 60 bpm	1755	− 0.03 (− 0.09, 0.03)	− 0.03 (− 0.09, 0.03)
RHR 60–69 bpm	1000	0.00 (reference)	0.00 (reference)
RHR 70–79 bpm	1015	0.01 (− 0.04, 0.07)	0.02 (− 0.03, 0.08)
RHR ≥ 80 bpm	520	− 0.10 (− 0.17, -0.03) ^†^	− 0.08 (− 0.15, − 0.01)*
Executive function z-score (n = 4253)			
RHR < 60 bpm	1742	− 0.03 (− 0.08, 0.03)	− 0.02 (− 0.08, 0.04)
RHR 60–69 bpm	992	0.00 (reference)	0.00 (reference)
RHR 70–79 bpm	1004	− 0.03 (− 0.08, 0.03)	− 0.02 (− 0.08, 0.04)
RHR ≥ 80 bpm	515	− 0.03 (− 0.11, 0.04)	− 0.02 (− 0.09, 0.05)

*MMSE* Mini-Mental State Examination, *RHR* resting heart rate

^a^N indicates the number of participants

^b^Model 1 was adjusted for age, sex, and education; and Model 2 was additionally adjusted for smoking, alcohol intake, body mass index, dyslipidemia, hypertension, diabetes, estimated glomerular filtration rate, cardiovascular morbidity, *APOE* genotype, anti-thrombotic agents, and cardiac agents

*P < 0.05

^†^*P* < 0.01

**Fig. 1:**
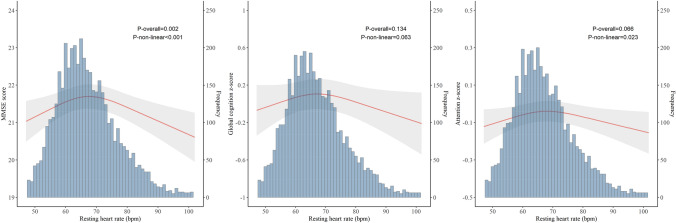
Associations of RHR with MMSE score and z-scores of global cognition and attention by restricted cubic spline models. *RHR* resting heart rate, *MMSE* Mini-Mental State Examination. The number of participants with available data was 4510 for MMSE score, 4180 for global cognitive z-score, and 4290 for attention z-score. Models were adjusted for age, sex, education, smoking status, alcohol intake, BMI, dyslipidemia, hypertension, diabetes, estimated glomerular filtration rate, cardiovascular morbidity, *APOE* genotype, anti-thrombotic agents, and cardiac agents.

The RCS modelling analysis suggested an inverted J-shaped association of RHR with MMSE score, such that both low and high RHR were associated with lower MMSE scores (*p*_overall_ = 0.002, *p*_non-linear_ < 0.001). Results on the associations of RHR with z-scores of global cognition and attention were overall similar to those with MMSE score (Fig. 1), while we did not detect any statistically significant association with z-scores of memory, verbal fluency, or executive function.

### Associations of RHR with LGI and ED biomarkers (n = 1386)

The RCS modelling analysis suggested linear associations of RHR with biomarkers of LGI and ED in the analytical sample 2 (Supplementary Fig. S2). Therefore, we used the linear regression models to examine the associations of RHR with these biomarkers. Increased RHR was significantly associated with higher concentrations of serum IL-6 (β coefficient = 0.19; 95% CI 0.14, 0.24), IL-8 (0.08; 0.02, 0.13), IL-10 (0.09; 0.04, 0.15), TNF-α (0.06; 0.01, 0.11), MCP-1 (0.09; 0.04, 0.15), ICAM-1 (0.16; 0.11, 0.22), and VCAM-1 (0.11; 0.06, 0.16) in multivariable-adjusted models (Table 3, Model 2).

**Table 3:** Association of serum biomarkers of low-grade inflammation and endothelial dysfunction with resting heart rate (per 10 bpm increase) (n = 1386)

Serum biomarkers^a^	β coefficient (95% confidence interval), serum biomarkers			
	Model 1^b^	*P* value	Model 2^b^	*P* value
IL-6	0.18 (0.13, 0.23)	< 0.001	0.19 (0.14, 0.24)	< 0.001
IL-8	0.07 (0.01, 0.12)	0.01	0.08 (0.02, 0.13)	0.004
IL-10	0.09 (0.04, 0.14)	< 0.001	0.09 (0.04, 0.15)	< 0.001
TNF-a	0.05 (0.00, 0.10)	0.04	0.06 (0.01, 0.11)	0.02
MCP-1	0.09 (0.04, 0.14)	< 0.001	0.09 (0.04, 0.15)	< 0.001
ICAM-1	0.16 (0.11, 0.21)	< 0.001	0.16 (0.11, 0.21)	< 0.001
VCAM-1	0.11 (0.06, 0.17)	< 0.001	0.11 (0.06, 0.16)	< 0.001

*IL-6* interleukin-6, *IL-8* interleukin-8, *IL-10* interleukin-10, *TNF-α* tumor necrosis factor alpha, *MCP-1* monocyte chemotactic protein-1, *ICAM-1* intercellular adhesion molecule 1, *VCAM-1* vascular cellular adhesion molecule 1

^a^Serum IL-6, IL-8, IL-10, TNF-α, ICAM-1, and VCAM-1 were log-transformed due to skewness of original data, and then converted to standard z-score; MCP-1 was directly converted to standard z-score

^b^Model 1 was adjusted for age, sex, and education; and Model 2 was additionally adjusted for smoking, alcohol intake, body mass index, dyslipidemia, hypertension, diabetes, estimated glomerular filtration rate, cardiovascular morbidity, *APOE* genotype, anti-thrombotic agents, and cardiac agents

## Discussion

The main findings from this population-based study of rural-dwelling non-demented older adults in China can be summarized into the following two aspects: (1) there was an inverted J-shaped association of RHR with global cognition and attention, such that a low RHR (< 60 bpm), especially an elevated RHR (≥ 80 vs. 60–69 bpm), was associated with a low score of attention and global cognition; and (2) an elevated RHR was linearly correlated with increased concentrations of serum cytokines and adhesion molecules. These findings suggested that an elevated RHR may be a valuable clinical marker for poor cognitive function in older adults, and that poor cognitive function and high RHR may share common pathways of systemic inflammation and endothelial injury.

Previously, the association of higher RHR (≥ 80 or 70–79 vs. < 60 bpm) with poor global cognition and dementia has been reported in some longitudinal studies [4, 5], but not in others [28, 29]. The inconsistency may attribute to survival bias, as evidence has shown that elevated RHR was associated with cardiovascular and all-cause mortality [3, 30, 31]. Our study extended the findings by revealing an inverted J-shaped association of RHR with global cognitive and attention scores. Attention is predominantly affected by microvascular lesions in the brain. In line with our results, data from UK Biobank showed a J-shaped association of RHR with all-cause dementia and vascular dementia, but not with Alzheimer’s disease [32]. In addition, low RHR (< 60 bpm) may reflect cardiovascular impairment [2], and the association of low RHR with poor cognitive function may be partly due to cerebral hypoperfusion resulting from low RHR. Collectively, these studies suggest that abnormal RHR and cognitive dysfunction may share common vascular pathophysiological process.

Previously, population-based cross-sectional studies showed that an elevated RHR was associated with some inflammatory biomarkers. For example, data from the US Multi-Ethnic Study of Atherosclerosis found that RHR was correlated with serum IL-6, hsCRP, and fibrinogen in middle-aged and older people free of CVD [20]. Besides, the Italian Aging in the Chianti Area study found that RHR was associated with IL-6 in older adults with sinus rhythm [33]. In addition, the Copenhagen City Heart Study demonstrated that RHR was related to hsCRP and fibrinogen in individuals with a median age of 56.2 years. [30] Our study extended the previous findings by showing that RHR was associated with serum cytokines (i.e., IL-6, IL-8, IL-10, TNF-a, and MCP-1) and adhesion molecules (i.e., ICAM-1 and VCAM-1) in a general population of older adults. LGI and ED may be the underlying pathophysiological process linking elevated RHR with worse cognition. Indeed, data from UK Biobank found that LGI biomarkers were associated with vascular dementia [34]. It has been suggested that ED may play a pivotal role in the pathophysiology of cognitive impairment [35]. Taken together, these results support the view that LGI and ED are involved, at least partly, in the association between high RHR and poor cognitive function in older adults.

Several potential mechanisms may underline the complex interrelationships between RHR, inflammation, endothelial injury, and cognitive function in older adults. First, altered RHR may be associated with cerebrovascular disorders (e.g., white matter lesions and clinical stroke) [28, 32], which in turn may affect cognitive function. Second, a high RHR may amplify the adverse effects of inflammation on cardiovascular system [36]. Given that plasma proinflammatory biomarkers were associated with worse cognitive function [15], an elevated RHR may enhance the adverse impact of LGI and ED on cognitive function. Third, systemic inflammation could affect RHR through imbalanced autonomic nervous system [3], which has been associated with mild cognitive impairment [37]. Finally, previous studies suggested that high RHR was associated with unhealthy lifestyle (e.g., physical inactivity, chronic stress, and insomnia) and metabolic diseases [20], which may be further linked with systemic inflammation and poor cognitive function. [13, 14, 20]. Given that RHR can be properly managed via regular exercise and use of medications (e.g., beta-blockers and ivabradine) and that lowering of heart rate could improve cardiovascular outcomes [38], further studies are warranted to assess whether non-pharmacological and pharmacological control of abnormal RHR would benefit cognitive function in older adults.

Our study targeted older adults who were living in rural communities of China with relatively low-income and who received no or limited education. Given that most studies have engaged urban populations in high-income countries, findings from our study would add to the current literature and contribute to health equity across socioeconomically diverse populations. Besides, by integrating profiles of serum cytokine biomarkers with extensive epidemiological, neuropsychological, and clinical data, we were able to explore the LGI and ED pathways linking RHR with global cognitive function and multiple cognitive domains. However, our study also has limitations. First, due to the nature of cross-sectional study, we cannot determine the causal relationship of RHR with LGI and ED biomarkers and cognitive function. The potential reverse causality should also be kept in mind when interpreting the observed cross-sectional associations. For example, individuals with subclinical cognitive impairment were less likely to participate in physical activity or exercise, which might affect RHR. Second, RHR was derived from a standard 12-lead ECG (a 10-s period), which may be less precise compared with other measurements such as 24-h Holter ECG, although it was easily accessible in the clinic settings and has been widely used in population-based studies [3, 5]. Third, although we have controlled for a wide range of possible confounders, we cannot rule out the residual confounding effects due to imperfect measurements of some confounders (e.g., self-reported lifestyle factors and health history). Finally, the study population was derived from only one rural area of western Shandong province in China, thus, it should be kept in mind when generalizing these findings to other populations.

In conclusion, our study revealed an inverted J-shaped correlations of RHR with global cognition and attention in rural older adults, with an elevated RHR (≥ 80 bpm) being associated with worse cognitive function. Furthermore, an elevated RHR was linearly associated with increased serum cytokines and adhesion molecules of systematic inflammation and endothelial injury. These findings contribute to the understanding of the complicated relationship of RHR with cognitive domains and inflammation in a general population of rural older adults.

### Supplementary Information

Below is the link to the electronic supplementary material.

Supplementary file1 (DOCX 284 KB)

## Data Availability

The datasets used and/or analyzed during the current study are available from the corresponding author upon reasonable request.
