# Supplementary material for: Resting heart rate, cognitive function, and inflammation in older adults: a population-based study
Source: Aging Clin Exp Res. 2023 Oct 29;35(11):2821–9. doi: 10.1007/s40520-023-02576-8 (PMC10628022; doi:10.1007/s40520-023-02576-8)
Supplement: Supplementary file 1 — Supplementary file1 (DOCX 284 KB) [file 40520_2023_2576_MOESM1_ESM.docx]

**SUPPLEMENTARY MATERIAL**

**Resting heart rate, cognitive function, and inflammation in older adults: a population-based study**

Ming Mao, MD,^1^ Rui Liu, MD,^1^ Yi Dong, MD, PhD,^2,3^ Chaoqun Wang, MD,^1^ Yifei Ren,^1^ Na Tian,^2,3^ Shi Tang, MD, PhD,^2,3^ Tingting Hou, MD, PhD,^2,3^ Lin Cong,^2,3^ Yongxiang Wang, MD, PhD,^2,3,4,5,6*^ Yifeng Du, MD, PhD,^1,2,3*^ Chengxuan Qiu, PhD^1,6^

^1^ Department of Neurology, Shandong Provincial Hospital, Shandong University, Jinan, Shandong, P.R. China;

^2^ Department of Neurology, Shandong Provincial Hospital affiliated to Shandong First Medical University, Jinan, Shandong, P.R. China;

^3^ Medical Science and Technology Innovation Center, Shandong First Medical University & Shandong Academy of Medical Sciences, Jinan, Shandong, P.R. China, 250021;

^4^ Institute of Brain Science and Brain-inspired Research, Shandong First Medical University & Shandong Academy of Medical Sciences, Jinan, Shandong, P.R. China;

^5^ Key Laboratory of Endocrine Glucose & Lipids Metabolism and Brain Aging in Shandong First Medical University, Ministry of Education of the People’s Republic of China, Jinan, Shandong, P.R. China;

^6^ Aging Research Center and Center for Alzheimer Research, Department of Neurobiology, Care Sciences and Society, Karolinska Institutet-Stockholm University, Stockholm, Sweden.

**CONTENTS**

**Supplementary Figure**

**Supplementary Figure 1.** Flowchart of the study participants.

**Supplementary Figure 2.** Associations of RHR with serum biomarkers of low-grade inflammation and endothelial dysfunction by restricted cubic spline models (n=1386).

MIND-China: baseline participants (age ≥65 years),

March-September 2018, n=5,246

Analytical sample 1, n =4,510

736 persons were excluded due to:

- Severe mental health problems (n=46);
- Dementia (n=302);
- Missing all cognitive assessments (n=144);
- Missing RHR (n=2);
- Missing covariates (n=242).

Analytical sample 2: a subsample with data on LGI and ED biomarkers, n=1,386

**Supplementary Figure 1. Flowchart of the study participants.**

Abbreviation: MIND-China, Multimodal Interventions to Delay Dementia and Disability in Rural China; RHR, resting heart rate; LGI, low-grade inflammation; ED, endothelial dysfunction.

**
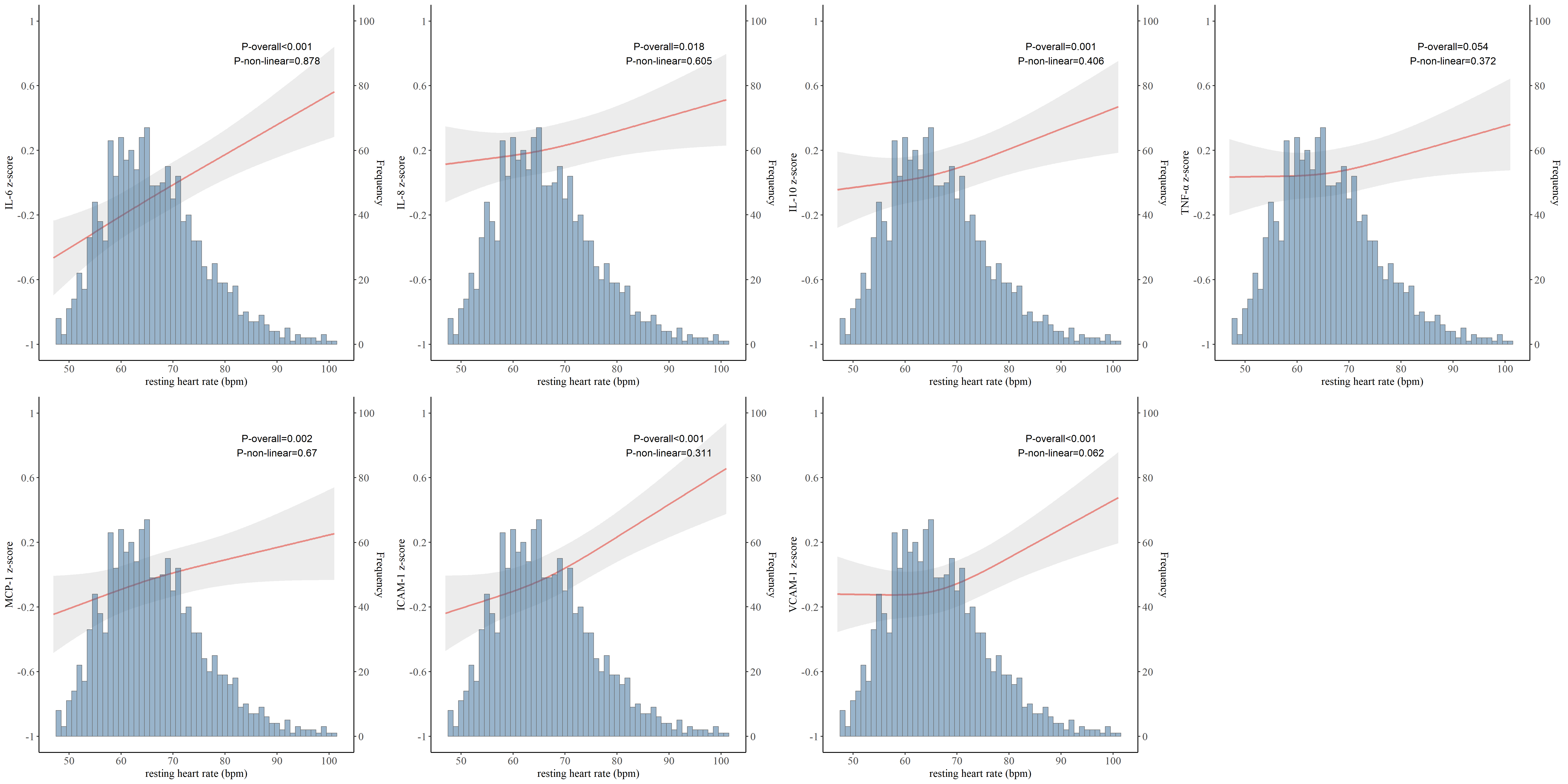
**

**Supplementary Figure 2. Associations of resting heart rate with serum biomarkers of low-grade inflammation and endothelial dysfunction by restricted cubic spline models (n=1,386).**

Abbreviations: IL-6, interleukin-6; IL-8, interleukin-8; IL-10, interleukin-10; TNF-α, tumor necrosis factor alpha; MCP-1, monocyte chemotactic protein-1; ICAM-1, intercellular adhesion molecule 1; VCAM-1, vascular cellular adhesion molecule 1.

Models were adjusted for age, sex, education, smoking status, alcohol intake, body mass index, dyslipidemia, hypertension, diabetes, estimated glomerular filtration rate, cardiovascular morbidity, *APOE* genotype, anti-thrombotic agents, and cardiac agents.

Serum IL-6, IL-8, IL-10, TNF-α, ICAM-1, and VCAM-1 were log-transformed due to skewness of original data, and then converted to standard z-score; MCP-1 was directly converted to standard z-score.
